# Reading a Story: Different Degrees of Learning in Different Learning Environments

**DOI:** 10.3389/fphar.2017.00701

**Published:** 2017-10-04

**Authors:** Anna Maria Giannini, Pierluigi Cordellieri, Laura Piccardi

**Affiliations:** ^1^Department of Psychology, Sapienza University of Rome, Rome, Italy; ^2^Department of Life, Health and Environmental Sciences, University of L’Aquila, L’Aquila, Italy; ^3^Neuropsychology Unit, IRCCS Santa Lucia Foundation, Rome, Italy

**Keywords:** multimedia, learning scenarios, verbal memory, visual memory, moral comprehension

## Abstract

The learning environment in which material is acquired may produce differences in delayed recall and in the elements that individuals focus on. These differences may appear even during development. In the present study, we compared three different learning environments in 450 normally developing 7-year-old children subdivided into three groups according to the type of learning environment. Specifically, children were asked to learn the same material shown in three different learning environments: reading illustrated books (TB); interacting with the same text displayed on a PC monitor and enriched with interactive activities (PC-IA); reading the same text on a PC monitor but not enriched with interactive narratives (PC-NoIA). Our results demonstrated that TB and PC-NoIA elicited better verbal memory recall. In contrast, PC-IA and PC-NoIA produced higher scores for visuo-spatial memory, enhancing memory for spatial relations, positions and colors with respect to TB. Interestingly, only TB seemed to produce a deeper comprehension of the story’s moral. Our results indicated that PC-IA offered a different type of learning that favored visual details. In this sense, interactive activities demonstrate certain limitations, probably due to information overabundance, emotional mobilization, emphasis on images and effort exerted in interactive activities. Thus, interactive activities, although entertaining, act as disruptive elements which interfere with verbal memory and deep moral comprehension.

## Introduction

Narrative language is a complex form of discourse that conveys information related to action, narrated events, and the internal states of the characters interacting in the story. Generally, narrative comprehension is an important step in human development and experience. Children’s ability to comprehend fictional narratives is related to three key aspects of the story: causal relationships in stories, goals and internal states of the characters in the stories, and integration of the different parts of the stories (Bruner, 1986; Bamberg, 1987; Hudson and Shapiro, 1991; Trabasso and Stein, 1997; van den Broek, 1997; Hickman, 2004; Rollo, 2007). The listener (or reader) has the expectation of logical coherence (cause and effect) between events (Graesser et al., 1980; Barthes, 1981). In general, narrative comprehension involves many perceptual and cognitive sub-processes, including perceiving individual words, parsing sentences, and understanding the relationships between characters (Wehbe et al., 2014). When a child reports events and facts from a story, he/she uses specific words that refer to internal states such as perceptions, emotions, and desires; very often, the child puts him/herself in the shoes of the main character (Baumgartner and Devescovi, 2001; Rollo, 2007; D’Amico et al., 2008). Children learn about characters, events and values through the spoken communication, miming and gestures of a narrator, or by reading texts directly in the simplest and most linear forms (texts are defined as *monomedia* when they use only writing or only illustrations, or as *bimedia* when writing and illustrations are combined). Trends in educational methods and in entertainment mean that children’s learning and memorization are limited to the abovementioned traditional methods. Currently, the development of “multimedia” techniques with the presentation of texts (and *hypertexts*) on computer screens (Jonassen, 1988; Landow, 1992; Furió et al., 2013; Butcher, 2014; Clark and Feldon, 2014) has extended educational methods. Critical approaches focus on the motivational aspects of multimedia procedures, which involve the implicit or explicit invitation to browse, explore and extend information, as well as to master the activities proposed by a computer. The satisfaction gained in this way makes the activity fun and pleasant according to some researchers (e.g., Furió et al., 2013), though it can also end up diverting children’s attention and affecting their learning and/or remembering of the proposed core content, particularly verbal information (for a critical review on motivational components see De Beni and Moè, 2000; Giannini, 2002; Mayer, 2014a). In particular, Kashihara et al. (2000) found that multimedia learners who are confronted with motivational elements may be distracted from information processing, with consequences for cognitive learning.

In studies which compare learning from traditional books and learning from computer screens it has been shown that text and illustrations may be more effective than narrated animations (Mayer et al., 2005). It is known, for instance, that externalizing a story improves children’s memory for that story (Glenberg et al., 2004) as well as internalizing a text through emotional expression and gesture (Noice and Noice, 2006). These results are also supported by neuroimaging studies that demonstrate that among the variety of brain regions that encode information about story characters, characters’ physical movements are represented in brain regions (i.e., the posterior temporal cortex/angular gyrus) that are implicated in the perception of biological motion (Grossman and Blake, 2001; Wehbe et al., 2014) and related to mental motor imagery. Other studies have investigated how the types of illustrations used influence how much children generalize after having read an illustrated book (Ganea et al., 2009; Tare et al., 2010). Children seem to learn more from illustrated books with realistic photographs or color drawings than simple line drawings (Simcock and DeLoache, 2006, 2008). However, the efficacy of illustrations and animations as tools for improving learning remains a controversial area; for example, conflicting results have been obtained by Lowe, who found learning facilitation with animations (Lowe and Schnotz, 2014; Ploetzner and Lowe, 2014; Lowe, 2015). Narrative memory presented in written or verbal form is enhanced by pictures (Levin and Lesgold, 1978; Brookshire et al., 2002; Carney and Levin, 2002) because exposure to information both verbally and pictorially provides redundant retrieval routes (Paivio, 1970, 1986). Pictures may also enhance attention to and comprehension or organization of material, or they may provide cues about important information in the text to keep activated. All of these factors may promote the formation of stronger, more elaborate and more organized memory trace (Gernsbacher, 1990; Levin and Mayer, 1993). Indeed, according to the seminal “dual coding” theory of Paivio (1991), there are two major systems engaged by the presentation of information: one related to verbal and linguistic stimuli and the other related to visual information and mental images. According some authors, multimedia presentation produces a beneficial effect on learning thanks to the “dual coding” hypothesized by Paivio. However, although the advantage of learning through multimedia is now accepted, there is still debate as to whether multimedia presentation is the optimal approach for giving instructions and learning content (Kalyuga et al., 1999; Higgins et al., 2009; Eitel et al., 2013; Arndt et al., 2015). Indeed, in some cases, multiple verbal and non-verbal presentations may add to the “cognitive load” of the user (Chandler and Sweller, 1991; Paas and Sweller, 2014). This especially applies to information presented redundantly. Sensory channel encoding has limited resources, and it is therefore necessary to avoid situations involving excessive cognitive load. According to cognitive load theory (Sweller, 1988), to facilitate changes in long term memory related to schema acquisition, it is necessary to reduce the cognitive load of learners to a minimum. One way to reduce cognitive load is by becoming increasingly familiar with the material. Familiarity alters the cognitive characteristics associated with the material. This promotes schema acquisition, making it easier to handle the material in working memory. Indeed, cognitive load results from several elements being held and manipulated simultaneously in working memory (Sweller, 1994). Unfortunately, working memory is a finite resource that can be overloaded; to overcome this limit, it is necessary to organize learned information into schemas. This organization, as mentioned above, allows more efficient learning. Learning is undoubtedly more lasting and durable when learners are cognitively engaged in the learning process (Bransford et al., 2000; Chinn, 2011). Accordingly, learning environments are most effective when they elicit effortful cognitive processing by guiding learners in actively constructing meaningful relationships rather than encouraging passive recording and storage of information (Craik and Tulving, 1975; Wittrock, 1992). This is the concept of “active development,” that recognizes the “importance of active participation of the student, who must necessarily act on the material presented through operations such as selection of the most meaningful information, the organization in an appropriate mental representation, and integration with the knowledge previously acquired, enabling the consolidation in long-term memory” (Wittrock, 1992). Some authors (Mayer and Moreno, 1998; Moreno and Mayer, 2000; Mayer, 2001, 2005, 2011, 2014b) have proposed a theoretical model based on this concept. The methods for achieving “active development” have been described by Mayer and are mainly related to respecting just a few rules. Among these rules is the spatial and temporal proximity of different signs: we learn better when corresponding words and pictures are presented physically close to each other and at the same time, or at least in sequence. This principle stems from dual coding theory (Paivio, 1971, 1991; Clark and Paivio, 1991; Mayer and Anderson, 1991), which suggests the importance of avoiding redundant forms that encumber attentive efforts and evaluating the message, especially by considering a criterion of consistency of information. Multimedia formats often do not take into account these considerations, resulting in reduced quality in the forms of learning, particularly in terms of narrative thought. Stories are a flexible language to interpret and talk about reality, but they still require structural continuity to be properly understood.

In the present study, we aimed to investigating narrative comprehension acquired through different learning environments (traditional illustrated books: TB; stories displayed on a PC screen enriched with interactive activities: PC-IA; or stories displayed on a PC screen but not enriched with interactive activities: PC-NoIA). In particular, we were interested in better understanding which story content (verbal content, visual details and moral) is advantaged when children read and incidentally learn a story through different learning environments that represent different modalities of presentation (i.e., written in a book or displayed on a PC screen with or without interactive elements). To achieve this purpose, we asked to a large sample of 7-year-old normally developing children to read three different stories acquired in these three different learning environments (TB vs. PC-IA vs. PC-NoIA). In this way, we explored incidental learning by asking children to interact with the story for a fixed time-limit and without asking them to explicitly learn the tale. We expected to observe differential effects on memory according to learning environment: we hypothesized deeper comprehension of the moral meaning as well as increased verbal memory in TB than in PC-IA and increased attention toward visuo-spatial details in PC-IA and PC-NoIA relative to TB. We also hypothesized that adopting two different PC learning environments would allow us to assess the effects of both interactive activities and reading through a PC screen, which requires different eye movement patterns in exploring the text than with traditional books. Furthermore, we reasoned that PC-NoIA may represent a middle ground between traditional learning methods and electronic devices. In such a way, we can investigate whether electronic devices *per se* induce children to pay attention to different features (verbal and/or non-verbal contents) even when interactive activities are not provided.

## Materials and Methods

### Participants

A large sample of 450 seven-year-old children, with both genders equally represented (233 girls, corresponding to 52.5%, and 211 boys, 47.5%) and without reported school difficulties, took part in the study. Knowledge of the three stories used as experimental material (i.e., Aladdin’s Lamp; The Three Little Pigs; and Adopting a Star) was an exclusion criterion, while one inclusion criterion was that all pupils had to be able to use a personal computer. Any participant who failed to meet the above-mentioned criteria was excluded from the experiment but was involved in a secondary task consisting of a pleasant reading which was not relevant to the purposes of the study. All participants attended primary schools or the Educational and Sport Centres of the Municipality of Florence (Italy). Each child was examined individually in an appropriate room of his/her school or center. Foreign children and those with learning difficulties and other neurodevelopmental diseases (as reported by their teachers or families) were not included in the study. None had primary visual or hearing impairments or had been diagnosed with a neurological condition.

The examiner subdivided participants in three groups consisting of three different learning environments: (i) individual traditional book reading (TB); (ii) individual reading of the story, displayed on the computer screen and interspersed with interactive activities (PC-IA); and (iii) individual reading of the story, shown on the computer screen but not interspersed with interactive activities (PC-NoIA). The precise sample for the TB condition was 78 girls and 70 boys, with 2 children’s gender not indicated; for PC-IA, there were 77 girls and 72 boys, with 1 child’s gender not indicated; and for PC-NoIA, there were 78 girls and 69 boys, with 3 children’s gender not indicated.

The three possible stories (Aladdin’s Lamp; The Three Little Pigs; and Adopting a Star) were equally distributed across the three learning environments. Furthermore, the difficulty and comprehensibility were balanced across the three stories.

The study was approved by the local ethical committee of the Department of Psychology, Sapienza University of Rome, in accordance with the Declaration of Helsinki. A signed consent form was obtained from parents and an assent from each child. Specifically, the consent obtained from the parents of all research participants was both informed and written.

### Materials

**FIGURE 1:**
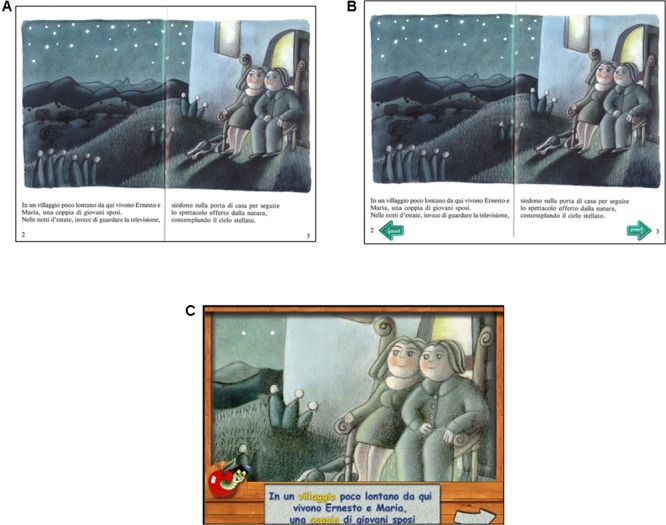
An example from the book “Adopting a Star” of the different modalities. **(A)** Presentation through traditional book (TB); **(B)** presentation through PC-NoIA; **(C)** presentation through PC-IA. The San Paolo publishing house has consented to the use of one picture from the book “Adottare una stella” (1993) by Massimo Mostacchi and illustrated by Monica Miceli, San Paolo Edizioni. The figure is being reproduced with the permission from the copyright holder.

We started by looking for editions of children’s stories in order to present identical texts and pictures in the three different learning environments (TB, PC-IA, and PC-NoIA). **Figure 1** reports an example taken from the book “Adopting a Star.” For each learning environment, corresponding to a different modality of story presentation, children read only one story. In the PC-NoIA condition the modality of story presentation was the same as in TB, and children were asked to move through the story using the mouse. The child chose to go forward or backward by clicking on two arrows, as if leafing through a book. In contrast, in the PC-IA condition various options were available, including listening to narration corresponding to the written text, with different voices for each character, as well as hearing animal noises and answering written questions by ticking boxes with the mouse.

We selected adaptations and re-editions of the following stories that had been published in Italy: (1) *La Lampada di Aladino* (“Aladdin’s Lamp”) published by Kyberkid, Città di Castello (Maestripieri, 2001); (2) *I Tre Porcellini* (“The Three Little Pigs”), published by Giunti, Florence (Escofet et al., 2000); and (3) *Adottare una Stella* (“Adopting a Star”), published by Edizioni S. Paolo, Cinisello Balsamo, Milan (Mostacchi, 1993).

The text in each story was between 650 and 1350 words, interspersed with color illustrations (ranging from 15 to 26 pictures). The illustrations were carefully checked to assess their relevance to the text, and we also ensured that the spatial arrangement of text and illustrations in the three versions used was more or less equivalent in terms of surface allotment (see **Figure 1** for an example).

The three stories did not differ in terms of verbal and non-verbal memory details reported independently from the learning environment (verbal details: *F*_2,441_ = 1.461, *p* = 0.233; $\eta_{p}^{2}$ = 0.006; observed power = 0.28; non-verbal details: *F*_2,441_ = 2.315, *p* = 0.100; $\eta_{p}^{2}$ = 0.008; observed power = 0.40). We also examined gender differences in emotional involvement, liking and interest arousal for each story (see **Table 1** for details), but no gender differences emerged.

**Table 1:** Gender effect for emotional involvement, liking and interest arousal for each story.

	Females		Males				
1st story	*M*	*SD*	*M*	*SD*	*F*	*P*	*$\eta_{p}^{2}$*
*Emotional*							
Involvement	8.29	2.63	7.59	3.19	2.819	0.141	0.015
Liking	8.26	2.80	7.81	2.84	0.907	0.343	0.006
Interest arousal	8.09	3.03	7.44	3.35	1.517	0.220	0.010
**2nd story**							
*Emotional*							
Involvement	8.62	2.25	8.67	2.08	0.015	0.903	0.000
Liking	8.45	2.33	8.31	2.29	0.154	0.695	0.001
Interest arousal	8.66	2.44	8.67	2.49	0.000	0.991	0.000
**3rd story**							
*Emotional*							
Involvement	7.94	2.25	7.94	2.21	0.000	0.987	0.000
Liking	7.94	2.18	7.96	2.32	0.003	0.956	0.000
Interest arousal	8.23	2.18	7.91	2.36	0.719	0.398	0.005

*M (means), *SD* (standard deviations) and analysis of variance (F, *P*, and $\eta_{p}^{2}$) are reported.*

For this reason, we merged the three stories in subsequent analyses.

Various interactive options were available, as well as the opportunity for motor activities by actually using the computer itself. Interactive activities included musical accompaniments, voices narrating the written text, different voices for each character in the story, animal noises, the movements of leading and secondary characters, animations of natural events (such as rain and storms, with voices naming them), ideas for games such as puzzles, mazes, revealing masked items, matching spoken and written words with pictures, riddles, constructions, and painting activities.

None of these activities were included in the PC-NoIA condition, which displayed only the written text and illustrations that appeared in the book format.

### Procedure

Interaction with each story in each learning environment was limited to approximately 20 min. The examiner did not require children to learn but rather to perform a silent reading of the stories. We preferred to investigate incidental learning derived from the three different learning environments, since this type of learning is more similar to everyday situations in which children of that age peruse written texts, enhancing the ecological validity of the study.

After reading the story, each child was individually required to complete a written test. This testing was unannounced, included 14 written questions and lasted approximately 25 min; the written answers were supplied immediately after children finished the story. Double-blind conditions were maintained throughout the experiment.

The degree of learning each child had achieved was assessed by collecting the written answers to 10 cued recall questions printed on a card. The first five questions concerned the child’s memory for important details of the story text, and the following five questions focused on the pictures. The complete set of questions for each of the three stories, subdivided into two categories (primarily verbal memory and primarily non-verbal memory) is given in the Appendix. The order of the questions was constant in each written test.

The questions were worded so that for children of this age, priority was given to visual non-verbal images rich in physiognomic properties (Werner, 1940); the written text was basically a support at this point. Indeed, the images further encouraged children to read the written text, thus forming an information flow through the integration of illustrations and words (Schnotz, 2014).

Levels of positive emotional involvement, appreciation and interest arousal were also assessed through three specific questions, each accompanied by a visually perceived evaluation scale ranging from 1 (minimum) to 10 (maximum) points, as shown in the Appendix. Obviously, any “No” answer yielded 0 points (although this outcome never occurred).

To assess whether and to what extent each child had been able to grasp the so-called “moral” of the story (its overall meaning and the ethical teachings each story conveyed) we added an open question: “Have you learned anything from the story?”

This last question also had to be answered in writing and was presented near the end of the 25-min individual written test session. The data afforded by answers to this question allowed us to study the frequency distribution of the answers according to two categories: (a) successful processing of a relevant moral; and (b) unsuccessful processing of a moral, perhaps with intrusion of or emphasis on irrelevant and/or heterogeneous contents.

It should be stressed that in each story the moral was relatively clearly stated. This simplified the scoring, which was based on the agreement of two out of three expert judges that evaluated the pertinence of each answer and the presence of errors, omissions, or intrusions.

## Results

**FIGURE 2:**
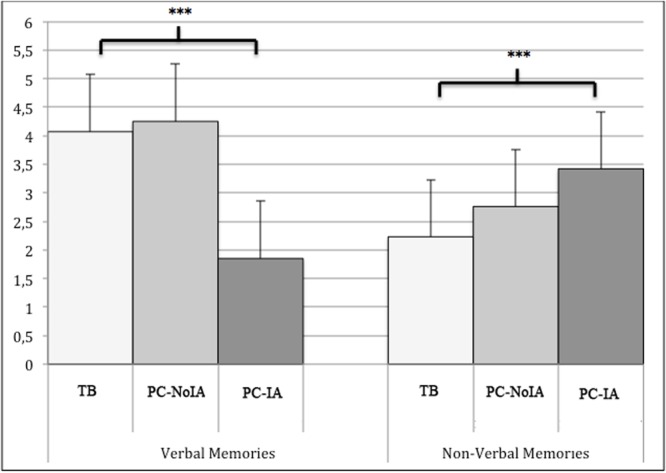
Mean scores for verbal and non-verbal memories in the TB, PC-NoIA, and PC-IA conditions.^∗∗∗^*p* < 0.001.

**FIGURE 3:**
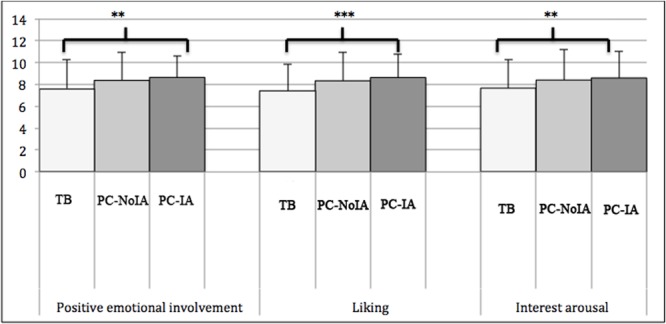
Mean scores for positive emotional involvement and interest arousal in the TB, PC-NoIA, and PC-IA conditions.^∗∗∗^*p* < 0.001, ^∗∗^*p* < 0.01.

**Figures 2, 3**, and **Tables 2–5** show statistics derived from the collected data. Recall performance was measured according to the number of correct and relevant memories for the first 10 questions (five relating to verbal memories and five relating to non-verbal memories), which were the same for all three stories in the three learning environments (**Figure 2**). An ANOVA showed that the three learning environments produced significant differences in verbal memory recall (*F*_2,441_ = 265.37; *p* < 0.001; $\eta_{p}^{2}$ = 0.54; observed power = 0.99). A Duncan *post hoc* test showed that TB produced higher performance (*p* < 0.05) for verbal details than did PC-IA. Verbal details reported for the TB and PC-NoIA conditions did not differ from each other. The TB and PC-NoIA conditions significantly differed from the PC-IA condition (*p* < 0.05) (**Table 2**).

**Table 2:** Overall recall scores for verbal and non-verbal memories.

Groups	*n*	Verbal memories		Duncan’s test^∗^	Non-verbal memories		Duncan’s test^∗^
		*M*	*SD*		*M*	*SD*	
TB	150	4.07	0.93	A	2.23	1.23	A
PC-NoIA	150	4.25	1.03	A	2.76	1.13	B
PC-IA	150	1.85	1.08	B	3.42	1.32	C
Analysis of Variance		*F*_2,441_ = 265.37 *p* < 0.001 *$\eta_{p}^{2}$* = 0.54			*F*_2,441_ = 37.29 *p* < 0.001 *$\eta_{p}^{2}$* = 0.12		

*M (means) and *SD* (standard deviations); n (number of participants). ^∗^Means with different letters (Duncan’s test) are significantly different (*p* < 0.05).*

**Table 3:** Overall scores for affective involvement.

Group	*n*	Positive emotional involvement		Duncan’s test^∗^	Liking		Duncan’s test^∗^	Interest arousal		Duncan’s test^∗^
		*M*	*SD*		*M*	*SD*		*M*	*SD*	
TB	150	7.61	2.69	A	7.41	2.45	A	7.63	2.64	A
PC-NoIA	150	8.37	2.53	B	8.33	2.59	B	8.40	2.78	B
PC-IA	150	8.64	1.99	B	8.63	2.16	B	8.59	2.43	B
Analysis of variance		*F*_2,441_ = 7.50 *p* < 0.01 *$\eta_{p}^{2}$* = 0.33			*F*_2,441_ = 10.53 *p* < 0.001 *$\eta_{p}^{2}$* = 0.42			*F*_2,441_ = 5.74 *p* < 0.01 *$\eta_{p}^{2}$* = 0.24		

*M (means) and *SD* (standard deviations); n (number of participants). ^∗^Means with different letters (Duncan’s test) are significantly different (*p* < 0.05).*

**Table 4:** Correlation matrix.

Learning environment	Memory	Emotional involvement	Liking	Interest arousal
TB	Verbal	0.193^∗^	0.116	0.048
	Non-verbal	-0.13	-0.102	-0.04
PC-NoIA	Verbal	0.169^∗^	0.172^∗^	0.289^∗∗∗^
	Non-verbal	0.199^∗^	0.102	0.171^∗^
PC-IA	Verbal	0.058	-0.037	-0.064
	Non-verbal	0.164^∗^	0.073	0.183^∗^

*^∗^*p* < 0.05, ^∗∗∗^*p* < 0.001.*

**Table 5:** Frequency distribution for responses concerning the morals of the stories.

	First story (Aladdin’s Lamp)		Second story (The Three Little Pigs)		Third story (Adopting a Star)	
	Moral	No moral	Moral	No moral	Moral	No moral
TB	37	13	24	26	32	18
PC-NoIA	16	34	31	19	21	29
PC-IA	20	30	8	42	11	39
	χ^2^ = 19.51		χ^2^ = 22.82		χ^2^ = 18.04	
	*p* < 0.001		*p* < 0.001		*p* < 0.001	

An ANOVA also showed a significant difference between the three learning environments for non-verbal memories (*F*_2,441_ = 37.29; *p* < 0.001; $\eta_{p}^{2}$ = 0.12; observed power = 0.91). *Post hoc* Duncan tests showed that recall performance was significantly higher in PC-IA (*p* < 0.05) relative to TB and PC-NoIA. Non-verbal memory recall was also significantly better also for the PC-NoIA condition (*p* < 0.05) relative to TB (**Figure 3**), but in the PC-NoIA condition non-verbal memory recall was significantly worse than that in the PC-IA condition (*p* < 0.05) (see **Table 2**).

Average scores for positive emotional involvement, liking, and interest arousal were calculated on the basis of answers supplied to the three evaluative questions, which focused on affective aspects of children’s experiences of the stories. Three separate ANOVAs were performed on positive emotional involvement, liking and interest arousal according to learning environment. Positive emotional involvement was significantly different for the three groups (*F*_2,441_ = 7.50; *p* < 0.01; $\eta_{p}^{2}$ = 0.33; observed power = 0.95). Duncan tests showed that these scores were significantly higher in the PC-IA group (*p* < 0.05; **Figure 2**). Scores were slightly lower in the PC-NoIA group and dropped significantly in the TB group (*p* < 0.05). Liking also significantly differed for the three learning environments (*F*_2,441_ = 10.53; *p* < 0.001; $\eta_{p}^{2}$ = 0.42; observed power = 0.98). Duncan tests showed that liking was significantly lower for the illustrated TB condition (*p* < 0.05). The three learning environments showed significant differences for interest arousal (*F*_2,441_ = 5.74; *p* < 0.01; $\eta_{p}^{2}$ = 0.24; observed power = 0.85). Duncan tests indicated a significantly lower score for the TB condition (*p* < 0.05) than the PC-IA and PC-NoIA conditions (**Table 3**).

To ascertain whether multimedia presentation truly made the activity of reading stories fun and pleasant, we performed a Pearson’s correlation analysis of verbal and non-verbal details reported in the three different learning environments (TB; PC-IA; and PC-NoIA) with emotions activated by the stories (emotional involvement, liking, and interest arousal). The analysis showed a significant positive correlation between the PC-IA condition and emotional involvement, but only for non-verbal details: specifically, for emotional involvement [*r*(147) = 0.164, *p* = 0.047] and liking [*r*(147) = 0.183, *p* = 0.041] but not for interest arousal. No significant correlations between emotional involvement and verbal details were observed. Interactive modalities had effects on the recollection of non-verbal but not verbal details. In contrast, in the TB condition emotional involvement and verbal details were positively correlated [*r*(145) = 0.193, *p* = 0.020]. Interestingly, the PC-NoIA condition demonstrated a positive correlation between verbal details and emotional involvement [*r*(152) = 0.169, *p* = 0.037], arousal interest [*r*(152) = 0.172, *p* = 0.034] and liking [*r*(152) = 0.289, *p* = 0.001]. Non-verbal details were also correlated with emotional involvement [*r*(152) = 0.199, *p* = 0.014], interest arousal [*r*(152) = 0.171, *p* = 0.035] and liking [*r*(152) = 0.289, *p* = 0.001]. Therefore, while the TB condition showed an effect only for verbal details and PC-IA for non-verbal details, the intermedia learning environment (PC-NoIA) exhibited significant correlations with both verbal and non-verbal details (see **Table 4** for the correlation matrix). As a consequence, we could hypothesize that emotional involvement, liking and interest arousal are more related to the device (reading on the PC vs. reading a traditional book) than performing additional activities during reading.

Concerning the question of the moral and teaching of each story, there were significant differences in the frequency of answers showing comprehension and retention of the relevant moral (i.e., the core meaning of the story) between groups (i.e., first story: χ^2^ = 19.51; *p* < 0.001; second story: χ^2^ = 22.82; *p* < 0.001; third story: χ^2^ = 18.04; *p* < 0.001). Specifically, the TB group was better at understanding the moral of the story (*p* < 0.01), whereas this frequency was lower in the PC-IA group. The PC-NoIA condition yielded intermediate results (see **Table 5**).

We observed qualitative differences in responses concerning the stories’ morals produced in the three learning environments. Here, we report examples of answers provided in the different learning environments. Specifically, children in the PC-IA group produced more irrelevant content, which generally referred directly to computer use, as opposed to story contents: for example, “I learnt to click …” (to use a mouse); “The arrows on the keyboard” (the cursors for turning the page); “That you turn over the page using the mouse”; “You can move things, trees, the dog, people, etc.”; “I learnt the maze”, and “The puzzle...” (or other story-related play activities). In contrast, children’s answers when they learnt through TB were more often pertinent, such as the following: “You mustn’t trust strangers”; “You must be careful when you suspect something”; “You shouldn’t trust strangers even when they promise you something gold”; “You must be righteous, good and never tell lies”; and “It is wrong to steal” (based on reading “Aladdin’s Lamp”). In the case of “The Three Little Pigs,” children in the TB condition responded: “You should work and be far-sighted in life”; “We must work well”; and “They should have built a brick house all together…”. For the story “Adopting a Star,” responses included: “I learnt that love between people is very important”; “Sometimes we should think of others and not just ourselves”; and “When you find something, you should always ask who it belongs to.”

## Discussion

Our main aim was to investigate differences in incidental learning produced by different learning environments (TB, PC-NoIA, and PC-IA). We hypothesized that differences in story presentation could induce differences in verbal and non-verbal memories as well as in deep comprehension of the story’s moral. With this intent, we analyzed different types of learning effects (verbal and visual memory) as well as moral comprehension, which involves deep comprehension of spiritual and ethical meanings that children will need to make choices and take actions reflective of universally accepted beliefs and values. The novelty of our study was to introduce an intermediate learning environment that did not require participants to perform interactive activities but displayed information on a PC screen, similarly to a book. The introduction of this further learning environment allowed us to better understand the effect of interactive activities on incidental learning. Furthermore, it allowed us to compare traditional teaching with teaching on a novel electronic device without adding any type of activities but only requiring participants to read the story displayed on the PC. Our results showed that children who had dealt with PC screen reading and performing interactive activities reached higher levels of positive emotional involvement, liking, and interest. This result is in line with other studies (e.g., Sun et al., 2008; Ahmadi Gilakjani et al., 2012; Furió et al., 2013, Furió et al., 2015) that found that students reported greater satisfaction and motivation when they learned through new technologies. Additionally, our results showed higher levels of positive emotional involvement, liking, and interest toward the stories presented on the PC with or without interactive activities, relative to TB. At the same time, our sample clearly verbally recalled fewer essential details of the narrative. In contrast, non-verbal memory was enhanced by the information conveyed through illustrations and/or animations, but interactive activities generally did not help in grasping the core meaning of the story, especially its ethical aspects. In other words, the way in which the story has been read contributes to the priority of the elements that are learnt and remembered. This priority was influenced by the appeal of specific elements and by the implicit role of distracting elements. However, in learning environments without interactive activities (PC-NoIA), recall of verbal details was comparable to that acquired through a traditional learning approach (TB), demonstrating that in multimedia presentation, it is very important how content is provided. Interestingly, the PC-NoIA condition, like the PC-IA condition, contributed to improve performance in recalling non-verbal details. However, the PC-NoIA condition exhibited stronger positive correlations than the TB condition with emotional involvement, liking and interest arousal both for verbal and non-verbal memory, suggesting that the new devices *per se* may enhance learning, especially when there are no added activities. Thus, PC presentation promotes attention toward non-verbal details even when they are not required to perform any type of activities. We speculate that this enhancement may be due to exploratory eye movements during reading that differ from those during TB reading. This difference could also be because we use a PC and not a tablet. Indeed, it is also possible that reading an eBook on a tablet is more similar to TB reading both in the way we hold the object by hand and in the ocular scanning performed by individuals. Another possibility may be related to the screen’s backlighting, which may increase performance on non-verbal details. Recent studies have demonstrated effects of luminous radiation from visual comfort to psychological and physiological wellbeing (e.g., Vandewalle et al., 2009; Ferlazzo et al., 2014).

Al-Qahtani and Higgins (2013) investigated the effects of e-learning, blended learning (which combines e-learning and traditional teaching), and classroom learning. They found a statistically significant difference between the blended learning method and the other two methods. However, these authors did not find any significant difference between the e-learning and traditional learning groups. Girard et al. (2013) analyzed studies that compared game-based learning tools with more traditional approaches and found that games had the same learning effect as traditional approaches. In contrast, our study seems to suggest that content requiring a deeper analysis of the text (such as the moral of a story) is reduced when children read the story on a PC, particularly when they are also involved in interactive activities.

A possible interpretation of our results is that interactive activities could act as distracters producing a reduction of attention and a greater focus on a perceptual level, which affects verbal learning. This effect could be attributed to both proactive and retroactive interference effects (i.e., interfering with verbal cognition learned both after and before the reading activity) (e.g., Bower and Mann, 1992; Mayer, 2001, 2005). An interference effect could be due to the need to continually coordinate visual perception and motor skills necessary to read by means of a PC, as well as to the psychological effort (in terms of cognitive load) that the PC interaction may involve. However, the inclusion criterion that children be familiar with PCs, should have reduced such interference, especially considering that all were competent with digital media and did not experience an increase in cognitive load while using a daily tool with which they were high familiar. Indeed, according to cognitive load theory (Sweller, 1988) when a learner is familiar with the material or with the environment, the familiarity effect reduces cognitive load and increases the activation of previously learned schema. However, according to Sweller (1994), learning environments with high interactivity (such as the PC-IA condition) may introduce extraneous cognitive load that can negatively interfere with learning. Another possible explanation is the split-attention effect (e.g., Ayres and Sweller, 2014) between verbal and the visual information as well as due to the entertaining activities children may perform while reading the story reading. These activities, although pertinent, may result in split attention. This explanation is also in line with evidence that reading the text without interactive activities (as in the PC-NoIA learning environment) produced equally detailed verbal recall.

In the present study, although all groups were able to comprehend the pertinent moral, participants that read the story on a PC screen more often gave inappropriate answers, as if to make up for gaps in essential memories. Children’s answers thus suggest that they gave priority to memorize irrelevant content, which generally involved computer use, as opposed to aspects of the content. Differences in the ability to identify and describe the moral of the story appeared to be related to differences in recalling verbally coded passages from the story and their interrelations, even if this interpretation does not explain why children exposed to the PC-NoIA condition did not obtain the same level as in the TB condition in comprehending the story’s moral, since their performance in remembering verbal details was comparable.

Our data demonstrate instead that using extensive interactive situations can adversely affect recall at a verbal level, thus significantly reducing specific memory performance. On the other hand, the latter increases for image recall on a non-verbal level. In other words, the congruence between memorized material and the recall task seems to matter most, along with possible interference or cooperation between different channels.

Karunanayaka et al. (2007) studied developmental trends in the neural substrates supporting narrative comprehension and found age-related differences in brain activity, which may reflect changes in local neuroplasticity. The authors performed a group-level independent component analysis (ICA) that allowed them to identify the involvement of the following right structures: primary auditory cortex, mid-superior temporal gyrus, the most posterior aspect of the superior temporal gyrus, hippocampus, angular gyrus and the medial aspect of the parietal lobule (precuneus/posterior cingulate). Furthermore, a left-lateralized network was also identified comprising the inferior frontal gyrus (including Broca’s area), inferior parietal lobule, and medial temporal lobe. This widespread cerebral network suggests hypotheses concerning functional segregation in Broca’s and Wernicke’s areas, the crucial role of the right hemisphere in narrative comprehension and increased left hemispheric dominance for language processing with age (Karunanayaka et al., 2007). Neuroimaging data stress the complexity of narrative comprehension, which involves a widespread network of brain areas in both the hemispheres. In line with this evidence, it is possible to hypothesize that differences in learning due to the learning environment in which text is learned may be related to activation of different brain areas. Thus, a traditional book elicits deeper comprehension of the story’s meaning, while an interactive book focuses on visual details that are probably elicited by activities that require children to pay attention to a story’s visual details. In contrast, written text forces a deeper processing of content, reducing distracting factors and requiring readers not to perform actions in response to the text but only to pay attention to its contents.

Additionally, our results provide practical observations that may be useful for educational techniques. Indeed, these findings contribute to better understanding of how technology interacts with and affects cognitive structures. From a practical point of view, if the aim is to memorize computer procedures and visual images in particular, then it is worth adopting a rich multimedia approach. If, on the other hand, we wish children to learn the core meaning of the story in the best possible way, then TB is still the recommended approach; alternatively, we recommend at least reducing the interactivity of the multimedia approach.

## Author Contributions

Conceived and designed the experiments: AMG, PC, and LP. Performed the experiments: PC. Analyzed the data: PC. Discuss the results and wrote the paper: LP, PC, and AMG.

## Conflict of Interest Statement

The authors declare that the research was conducted in the absence of any commercial or financial relationships that could be construed as a potential conflict of interest.
