# Supplementary material for: Reading a Story: Different Degrees of Learning in Different Learning Environments
Source: Front Pharmacol. 2017 Oct 4;8:701. doi: 10.3389/fphar.2017.00701 (PMC5649183; doi:10.3389/fphar.2017.00701)
Supplement: Supplementary file 1 [file Data_Sheet_1.docx]

**Appendix**

**1.**

**List of the 10 questions for each of the three stories used in the experiment**

| 1. Story: *Aladdin’s Lamp*: 1. What job does Aladdin’s mother do? 2. What does Aladdin bring the sultan? 3. What is the merchant looking for? 4. What does the princess put in the magician’s tea? 5. How long do the celebrations last? 6. What colour are Aladdin’s clothes? 7. What colour is the genie’s turban? 8. Where is the sultan sitting? 9. What does the princess bring the magician on a tray? 10. What colour is Aladdin’s turban later on in the story? |
| --- |
| 1. Story: *The Three Little Pigs*: 1. Where do the three little pigs live? 2. Where do they run away to? 3. What does the oldest little pig use to build his house? 4. Where do the little pigs pick the apples? 5. What does the wolf use to get onto the roof? 6. What colour is the mother’s dress? 7. What colour are the flowers in the clearing? 8. What is in front of the youngest little pig’s home? 9. What animal is in the bucket? 10. What is leaning against the brick house when the wolf tries to get in? |
| 1. Story: *Adopting a Star*: 1. What do Ernesto and Maria do on summer nights? 2. Where does Ernesto go in the morning? 3. Where do Ernesto and Maria go with the girl? 4. Why are the stars in such a hubbub? 5. What do the stars decide at the end of the story? 6. What is next to Maria and Ernesto while they are looking at the stars? 7. What is next to the house? 8. What does Ernesto have over his shoulder when he goes to work? 9. What is in the town square? 10. What colour is Stella’s ball? |

Note: Question categories: for each fable, questions 1 to 5 refer to mainly verbal memories; 6 to 10 refer to mainly non-verbal memories (spatial relations, positions, colours).

**2.**

Questions and evaluation scales to assess affective aspects. The scale ranges from 1 (minimum) to 10 (maximum) points

| Did you enjoy yourself? | 1 | 2 | 3 | 4 | 5 | 6 | 7 | 8 | 9 | 10 |
| --- | --- | --- | --- | --- | --- | --- | --- | --- | --- | --- |
| Did you like the story? | 1 | 2 | 3 | 4 | 5 | 6 | 7 | 8 | 9 | 10 |
| Would you like to read more? | 1 | 2 | 3 | 4 | 5 | 6 | 7 | 8 | 9 | 10 |
